# Systematic review and meta‐analysis of retention and disengagement after initiation on antiretroviral therapy in low‐ and middle‐income countries after the introduction of Universal Test and Treat policies

**DOI:** 10.1002/jia2.70026

**Published:** 2025-09-05

**Authors:** Amy Zheng, Emma M. Kileel, Alana T. Brennan, David B. Flynn, Sydney Rosen, Matthew P. Fox

**Affiliations:** ^1^ Department of Epidemiology Boston University School of Public Health Boston Massachusetts USA; ^2^ Department of Global Health Boston University School of Public Health Boston Massachusetts USA; ^3^ Health Economics and Epidemiology Research Office, Faculty of Health Sciences University of the Witwatersrand Johannesburg South Africa; ^4^ Department of Medical Sciences and Education Boston University Chobanian and Avedisian School of Medicine Boston Massachusetts USA

**Keywords:** antiretroviral therapy, HIV, lost to follow‐up, retention, systematic review, universal test and treat

## Abstract

**Introduction:**

We previously published a systematic review evaluating retention in care after antiretroviral therapy initiation among adults in low‐ and middle‐income countries from 2008 to 2013. This review evaluates retention after the implementation of Universal Test and Treat (UTT) in 2015.

**Methods:**

We searched PubMed, ISI Web of Science, Cochrane Database of Systematic Reviews and EMBASE for studies published 1 January 2017, through 31 December 2024 and searched conference abstract repositories from AIDS, IAS and CROI from 2015 to 2024. Retention for each study was estimated using (1) simple averages and (2) interpolated for missing time points through the last reported time point. Our outcomes were all‐cause attrition and retention. We estimated retention rates using a generalized linear mixed model (GLMM) with a logit distribution using interpolated data.

**Results:**

Seventy studies met our inclusion criteria. Most studies came from Africa, with very few from Europe and Asia. Few studies reported retention past the first 12 months following treatment initiation. Across all studies, we estimated simple average retention without interpolation of missing time points to be 72.6% at 12 months, 75.2% at 24 months, 67.7% at 36 months and 64.8% at 48 months. Utilizing a GLMM model, we estimated retention to be 79.6% at 12 months, 81.2% at 24 months, 75.6% at 36 months and 72.8% at 48 months. Whereas in our prior 2015 review, we estimated retention rates to be 86.0% at 12 months, 79.0% at 24 months, 75.0% at 36 months, and 69.0% at 48 months. These results generally reflect retention at the initiating facility and omit the effect of unreported transfers.

**Discussion:**

Retention in care at 36 months was estimated to be between 67% and 75%. Compared to results from our prior review, retention is largely similar in the post‐UTT era. Further research evaluating retention in other geographic areas (i.e. Latin America and the Caribbean, Europe, and Asia) is needed.

**Conclusions:**

Attrition after the first 2 years in treatment remains a concern, and concerted efforts should be made to ensure patients remain engaged in care over their lifetime. The impact of PEPFAR's recent cuts needs to be evaluated further to understand the effect it may have on long‐term retention.

## 1 INTRODUCTION

Despite the dramatic success of HIV treatment programmes globally, with nearly 30 million people living with HIV (PLWH) reported to be on antiretroviral therapy (ART), high rates of treatment interruption and disengagement from HIV care persist [1]. Lifelong retention in HIV care remains a challenge worldwide [2, 3, 4, 5]. Numerous reviews of adults and adolescents in HIV treatment programmes have documented varying levels of retention in care over time, as well as a range of barriers to short‐ and long‐term retention [6, 7, 8, 9, 10, 11, 12]. These efforts are further complicated by undocumented transfers (i.e. silent transfers) and/or deaths and patients who disengage and re‐engage in care at later points in time, which can lead to underreporting of retention [13]. Our own 2015 review found that retention at 36 months following ART initiation averaged between 65% and 70% [8]. Since retention in care is a prerequisite for viral suppression, which is the ultimate goal of HIV treatment, achieving and sustaining high rates of retention in care is essential.

Since our 2015 review, the landscape of HIV care and treatment has changed dramatically. The most significant development was the adoption of the Universal‐Test‐and‐Treat (UTT) strategy in 2015–2016 [14]. Under UTT, all individuals testing positive for HIV are eligible for immediate ART initiation, rather than waiting for a specific point of disease progression, as in the past [14, 15]. In addition, newer regimens featuring dolutegravir, known for reduced toxicity, have been introduced alongside significant programmatic advancements, including same‐day ART initiation and differentiated service delivery (DSD) models for treatment [14, 15, 16, 17, 18]. These changes have been aimed, in part, at improving overall rates of retention in HIV care [14, 15, 18, 19, 20, 21, 22]. Studies evaluating the impact of UTT on retention have suggested that UTT may lead to short‐term improvements in retention, but the long‐term effects are less clear.

A recent review (2020−2023) focusing solely on sub‐Saharan Africa found no difference in retention rates before and after UTT in the first 12 months post‐ART initiation, but did not consider longer time periods or other regions [7]. Another review found no notable differences in retention between patients enrolled in DSD models compared to patients enrolled in conventional models of care [10]. Many of these reviews have been limited in scope, looking only at specific care models, drug regimens, regions or populations [10, 23, 24]. Few studies, if any, have explicitly focused on comparing pre‐ and post‐UTT retention to determine whether the policy change—marked by a substantial shift in overall ART patient population characteristics, including higher average CD4 counts and fewer symptomatic patients—was associated with improvements in retention. As a number of publications have reported retention in routine care among specific cohorts in the years since UTT was adopted, it is an opportune time to conduct an updated review of the literature [1, 14, 21].

To evaluate changes in retention on ART in routine care settings since the implementation of UTT, we conducted a systematic review and meta‐analysis for low‐ and middle‐income countries (LMICs) globally. Our goals were to: (1) estimate all‐cause attrition from ART programmes in LMICs in the UTT era; (2) determine how attrition varies across regions; (3) evaluate what proportion of attrition can be attributed to death, transfer out of care and disengagement from care; and (4) describe how retention trends in the UTT era differ to pre‐UTT using findings from our pre‐UTT retention review in 2015. We also compare our findings to those of other recent reviews that have considered only specific subsets of the populations we include.

## 2 METHODS

### 2.1 Search strategy and selection criteria

This systematic review and meta‐analysis followed the Preferred Reporting Items for Systematic Reviews and Meta‐Analyses (PRISMA) guidelines and is registered with PROSPERO (396758). Ethics review was not required as no human subject data were used.

We developed our search strategy in consultation with a medical librarian (DBF). Inclusion and exclusion criteria are listed in Table 1. We limited the review to studies that enrolled participants newly initiating ART and started at ART initiation and provided enough information to estimate the rate of all‐cause attrition for at least one of the following time points: 6, 12, 18, 24, 36, 48, 60, 72, 84 and/or 96 months after ART initiation or at the end of the median follow‐up period. We excluded randomized trials. If observational studies evaluated the impact of an intervention but did not seek to alter retention for the comparison arm, only data from the comparison arm (i.e. those who did not receive the intervention) were included in this review. We included all World Bank‐defined LMICs [25]. We made no exclusions based on data quality. When multiple studies reported on a single cohort, we selected the one with the longest follow‐up. While a commonly adopted definition of loss to follow‐up (LTFU) is PEPFAR's (e.g. a patient is lost if they have not returned for more than 28 days), and many programmes were PEPFAR supported, we accepted each study's own definitions of attrition and retention, as we did not have access to the primary data to apply a consistent definition [26]. We were unable to distinguish between initiation of ART‐naive clients and re‐initiation of those returning to care after an interruption, as this characteristic is rarely reported accurately and instead relied on whether the study reports participants to be newly initiating ART [27].

**Table 1:** Inclusion and exclusion criteria used to determine which studies to be included for analysis in a systematic review of retention in care among those on antiretroviral therapy in low‐ and middle‐income countries from 2015 to 2024

Criterion	Inclusion	Exclusion
Geographic range	Low‐ and middle‐income countries as defined by the World Bank	High‐income countries as defined by the World Bank
Topic	Treatment of HIV with highly active antiretroviral therapy (ART) (≥3 drug combination, HIV‐1 only)	Anything other than treatment of HIV‐1 with ≥3‐drug ART
Language	English	Not English
Design	Observational cohort, intention to treat. Must report on outcomes for all participants who discontinued treatment for any reason.	Does not observe a cohort or reports only treatment results or treatment plus mortality results; clinical trial, including Phase 3 trials.
Setting	Standard service delivery in any sector (public, non‐governmental, private) that treats the general population.	Research or any other non‐standard setting in which national guidelines are not followed.
Guidelines in effect	Universal test and treat	Restrictions on treatment eligibility due to CD4 count or disease stage
Population	Adults (15+) who initiated or re‐initiated first‐line ART, including both general populations and special populations such as men who have sex with men, prisoners, drug use, sex workers, transgender persons.	Subsets of general treatment‐eligible population (e.g. participants known to have drug resistance or specific baseline CD4 counts).
Observation period	Must start at ART initiation or re‐initiation, with minimum median follow up of 6 full months (26 weeks); median follow‐up period must be clearly specified or able to be calculated from information provided; dates of observation period must be reported.	Already on ART at study enrollment. Median follow up of less than 6 full months (<24 weeks) or not specified and not able to be calculated from information provided.
Minimum information included	Must report all‐cause attrition rate at one of 6, 12, 18, 24, 36, 48, 60, 72, 84 or 96 months or at the end of the median follow‐up period or provide information needed to calculate one of these rates.	Does not report all‐cause attrition rate at least one of 6, 12, 24, 36, 48, 60, 72, 84 or 96 months or at the end of the median follow‐up period (and none of these rates can be calculated from information reported) or does not report dates of period of observation.
Data collection	2015−2024	Prior to 2015 or after 2024

### 2.2 Search procedures

Two reviewers (AZ and EMK) independently searched PubMed, ISI Web of Science, Cochrane Database of Systematic Reviews and EMBASE (full list of search terms is given in Supplementary Materials) from 1 January 2017, to 31 December 2024 for eligible publications and abstracts from AIDS (2016, 2018, 2020, 2022, 2024), IAS (2015, 2017, 2019, 2021, 2023) and CROI (2015−2024) conferences. Search terms were developed based on the following terms: “attrition,” “engagement,” “disengagement,” “interruption,” “non‐adherence,” “retention” and “loss to follow up” (Table S1). We conducted a hand search to capture articles not MeSH‐indexed and reviewed the reference lists of articles originally selected for data extraction to identify any studies missed [28]. 1 January 2017 was chosen as the starting date because implementation of UTT began in 2015, and studies published prior to 2017 were likely to report pre‐UTT data.

Each citation (title and abstract) was screened in duplicate [29] for eligibility for full‐text review using the criteria in Table 1. Both title/abstract and full text screening were done by two reviewers (AZ/EMK) independently, with any discrepancies resolved by a third reviewer (MPF). Reviewers used a standardized form to extract all relevant information, such as definitions of LTFU, follow‐up time and percent retained at each time point.

Study quality was evaluated using a modified version of the Joanna Briggs Institute (JBI) Critical Appraisal Tool for Prevalence Studies [30]. The following seven questions were used: (1) Was the cohort well‐defined? (e.g. Question 1 [Was the sample frame appropriate to address the target population] and Question 2 [Were study participants sampled in an appropriate way] from the original tool); (2) Was the definition of LTFU clear and well‐defined?; (3) Were LTFU outcomes clearly reported?; (4) Did the study differentiate between “Alive and on ART” and “Retained in Care”?; (5) Did the study differentiate between transfer, death, LTFU and so on?; (6) Was follow‐up time reported, assumed or calculated? (e.g. Question 8 [Was there appropriate statistical analysis] from the JBI tool); (7) Were valid methods used for the identification of the condition? (e.g. Question 6 unmodified from the JBI tool). Questions 2–5 were based on the following questions from the JBI tool: Question 4: Were the study subjects and the setting described in detail; Question 5: Was the data analysis conducted with sufficient coverage of the identified sample; and Question 7: Was the condition measure in a standard, reliable way for all participants. Questions 1–5 had the following response options: “Yes,” “No,” “Unclear” and “Not Applicable” [31]. Question 6 had the following responses options: “Reported,” “Assumed,” “Calculated” or “Not Applicable.” Table S2 provides an overview of the modifications made to the JBI tool for this systematic review. As the JBI tool does not provide point values to each question, we assigned point values to each response option to calculate an overall score with a maximum score of 13 and a minimum score of −7, with a higher score indicating higher quality. Questions 3 (Was the sample size adequate) and 9 (Was the response rate adequate, and if not, was the low response rate managed appropriately) from the JBI tool were not applicable to this quality evaluation, as we were evaluating incident outcomes.

### 2.3 Outcomes and data analysis

To make our results comparable to those of our earlier review, we applied the same methodology, to the extent possible, in this analysis [8, 9]. For each study, we report overall attrition (death + LTFU) from care at each time point reported in the study and report reasons for attrition, including death, LTFU and transfer to another facility. Retention is defined as 1 − % attrition. We report median follow‐up time for each study. If a study did not report median follow‐up, but all participants had the potential to complete the maximum reported follow‐up time, and retention was ≥50% at the end of follow‐up, then median follow‐up time can be assumed to be equal to the maximum reported follow‐up time. For example, if maximum follow‐up was 48 months, all participants were eligible to be followed for 48 months, and 80% were retained at 48 months, then the median follow‐up time would be considered 48 months. If everyone did not have the potential to complete the maximum reported follow‐up, we took the midpoint of the reported minimum and maximum follow‐up periods. For example, if a study enrolled participants from 2016 to 2018 and the follow‐up ended in 2020, some participants would only have the potential for 24 months of follow‐up, while others would have the potential for 48 months. In this scenario, we assumed the median follow‐up to be 36 months. Two studies reported mean follow‐up time, and we used that in place of the median [32, 33].

We estimated mean retention at site at 6, 12, 18, 24, 36, 48 and 60 months in five ways: (1) using simple averages of retention reported by each study; (2) using averages weighted by sample size for each cohort only at time points reported by each study; (3) using weighted averages and interpolated data; (4) using a generalized linear mixed model (GLMM) with a logit distribution and interpolated data as it accounts for the sample size of each cohort and for within‐ and between‐study variation which the other approaches do not. Our primary approach was the GLMM. We interpolated any missing period where possible, assuming a linear decline. For example, if a study reported outcomes at 24 months but not at 6 and 12 months, we interpolated 12‐month retention as the average of 24‐month retention and 100% and interpolated 6‐month retention as the average of the interpolated 12‐month retention and 100%. We generated bootstrapped 95% confidence intervals (CIs) utilizing the GLMM estimates with 10,000 samples [34, 35]. We stratified countries into the following regions: Europe and Asia, Latin America and the Caribbean, and Africa to evaluate how retention varied across regions. As the rollout of UTT in 2015 may have been limited with full adoption and implementation by most countries, especially in sub‐Saharan Africa, from 2016 onwards, we conducted a sensitivity analysis, excluding any study that used data from 2015. To describe trends in the UTT context and how retention rates differ in the post‐UTT era to the pre‐UTT era, we plotted the retention rates from this review against the retention rates from our prior 2015 systematic review [8].

In our prior reviews, we evaluated retention at each time point using a lifetable analysis and Kaplan‐Meier curves. In the current review, however, we found that a majority of studies were limited to shorter follow‐up periods (e.g. the first 12 months only), unlike in previous reviews. Because of this, a lifetable analysis would result in many study participants being censored and would generate a falsely low estimate of retention in care. We, therefore, omitted this type of analysis in this review.

Because not all studies reported retention for the same time points, we conducted a sensitivity analysis modelling three scenarios for attrition based on the interpolated data. The best‐case scenario assumed no attrition after the last point reported up to 72 months. The worst‐case scenario assumed attrition continued in the same linear trend as observed. The midpoint scenario was an average of the best‐case and worst‐case scenarios.

## 3 RESULTS

### 3.1 Eligible articles and study populations

**Figure 1:**
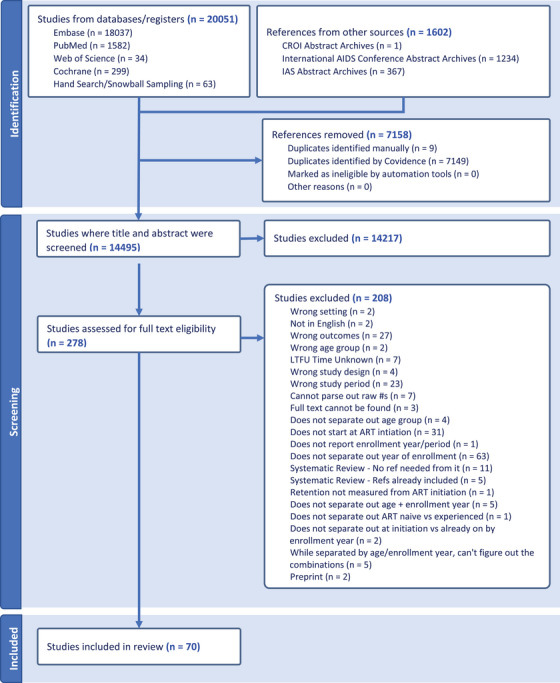
Flowchart of studies evaluated to be included in a systematic review and meta‐analysis of retention in care among those on antiretroviral therapy in low‐ and middle‐income countries from 2015 to 2024. Abbreviations: ART, antiretroviral therapy; CROI, Conference on Retroviruses and Opportunistic Infections; IAS, International AIDS Society; LTFU, lost to follow‐up.

Our search yielded 14,495 unique articles and abstracts. Of these, 70 met the inclusion criteria (66 articles and 4 abstracts; Figure 1). These studies reported on 70 cohorts and 483,827 individuals (Table S3). As in prior reviews, the majority (84.3%) of studies reported on cohorts from sub‐Saharan Africa. Of the 60 studies in Africa, 23.3% (20.0% of all studies) were from South Africa. The other 11 studies came from the Caribbean (*n* = 1), Asia (*n* = 9) and Europe (*n* = 1). We found no eligible studies from Latin America and were unable to evaluate retention outcomes in this region.

Among people initiating ART, 61.3% were female, and the majority were in their 30s. The median CD4 cell count at initiation ranged from 200 to 300 cell/mm^3^. People initiating in later years (2017−2019) had slightly higher median CD4 counts than those reported from earlier years (2015−2017). Most studies (87.3%) reported on retention for only the first 12 months after ART initiation (Table S3).

Definitions of disengagement from care or LTFU ranged from not returning to the clinic within 2 months of a scheduled visit (clinic or drug refill) to no return 9 months after the initial visit. The most common definition of disengagement was being late for the last scheduled visit or medication pickup by more than 90 days. For studies that made a distinction between LTFU and treatment stoppage, the most common definition for treatment stoppage was that the patient was known to be alive but had stopped ART treatment for any reason. For the three studies that reported on treatment interruptions, Dorward et al. defined treatment interruption as not having a visit for 180 days but then having a visit and restarting treatment [33]; Tlhajoane et al. defined interruptions as returning to care after an absence of >90 days [36]; and Ibiloye et al. reported the number of patients who experienced a treatment interruption but did not provide a definition [37]. All definitions used by the included studies are presented in Table S4.

### 3.2 Retention outcomes

Table 2 presents the unweighted retention at each time point for each study, weighted averages for overall retention, and a weighted average utilizing interpolated data. Across all studies, simple average retention with no interpolation was 72.6% at 12 months, 75.2% at 24 months, 67.7% at 36 months and 64.8% at 48 months, with substantial heterogeneity across individual studies and countries (Tables 2 and 3). Few studies reported outcomes beyond 36 months (*n* = 5). Results from the weighted average and weighted average using interpolated data were similar, and trends were consistent (Table 2).

**Table 2:** Retention of participants at 6, 12, 18, 24, 36, 48, 60 and 72 months after ART initiation calculated according to the data reported by each study included in a systematic review of retention in care among those on antiretroviral therapy in low‐ and middle‐income countries from 2015 to 2024

Reference	*N*	Percentage of participants retained at month:									
		6	12	18	24	36	48	60	72	84	96
Ahmed 2020 [38]	988	86.1	79.3	−	−	−	−	−	−	−	−
Alhaj 2019^a^ [39]	1492	−	74.7	−	−	−	−	−	−	−	−
Atuhaire 2022 [40]	275	85.1	78.9	−	75.3	−	−	−	−	−	−
Avalos 2019 [41]	1523	−	95.9	−	−	−	−	−	−	−	−
Benzekri 2022 [42]	207	−	59.9	−	−	−	−	−	−	−	−
Byamukama 2022 [32]	976	−	−	−	−	−	−	89.6	−	−	−
Cassidy 2023 [43]	1553	74.0	66.0	−	−	−	−	−	−	−	−
Chauke 2020 [44]	367	86.1	59.6	−	−	−	−	−	−	−	−
Dorward 2020 [33]	4952	−	68.3	−	−	−	−	−	−	−	−
Dumchev 2022 [45]	1057	−	90.5	−	−	−	−	−	−	−	−
Eamsakulrat 2022 [46]	270	−	84.1	−	−	−	−	−	−	−	−
Ntamatungiro 2021 [47]	902	−	59.6	−	−	−	−	−	−	−	−
Eshiwani 2018 [48]	167	72.5	−	−	−	−	−	−	−	−	−
Harooni 2022 [49]	124	−	94.3	−	−	−	−	−	−	−	−
Hirasen 2020 [50]	1143	−	57.3	−	−	−	−	−	−	−	−
Hoang 2022 [51]	4892	−	92.2	−	−	−	−	−	−	−	−
Ibiloye 2018 [37]	710	63.9	−	−	−	−	−	−	−	−	−
Ibiloye 2021 [52]	3495	−	63.5	−	55.4	51.2	46.7	−	−	−	−
Izudi 2022 [53]	9952	−	19.1	−	−	−	−	−	−	−	−
Jamieson 2021 [54]	32,197	−	−	95.7	−	−	−	−	−	−	−
Januraga 2018 [55]	606	75.4	−	−	−	−	−	−	−	−	−
Johansson 2021 [56]	2043	−	69.6	−	−	−	−	−	−	−	−
Kimanga 2022 [57]	8592	−	76.7	−	−	−	−	−	−	−	−
Lilian 2020 [58]	32,290	69.7	−	−	−	−	−	−	−	−	−
Makurumidze 2020 [59]	3636	−	−	−	91.8	−	−	−	−	−	−
Matare 2020 [60]	3971	76.7	72.3	−	−	−	−	−	−	−	−
Matthews 2020 [61]	437	−	94.5	−	−	−	−	−	−	−	−
Mayasi 2022 [62]	11,281	89.0	83.0	−	76.0	69.0	67.0	−	−	−	−
Mshweshwe‐Pakela 2020 [63]	710	67.2	−	−	−	−	−	−	−	−	−
Nshimirimana 2022 [64]	29,829	−	97.7	−	93.5	87.3	81.0	75.9	74.7	−	−
Onoya 2020 [65]	297	−	74.4	−	−	−	−	−	−	−	−
Opio 2019 [66]	646	−	55.6	−	−	−	−	−	−	−	−
Opito 2020^b^ [67]	536	−	78.7	−	−	−	−	−	−	−	−
Pillay 2019 [68]	120	69.0	−	−	−	−	−	−	−	−	−
Rogers 2021 [69]	423	−	−	−	73.0	−	−	−	−	−	−
Romo 2022 [70]	3563	−	71.9	−	−	−	−	−	−	−	−
Ross 2019 [71]	1082	51.7	−	−	−	−	−	−	−	−	−
Seekaew 2019 [72]	180	90.0	92.9	−	−	−	−	−	−	−	−
Seekaew 2021 [73]	1904	75.0	75.1	−	−	−	−	−	−	−	−
Ssempijja 2020 [74]	1305	−	84.0	−	−	−	−	−	−	−	−
Stafford 2019 [75]	2652	−	59.5	−	−	−	−	−	−	−	−
Stockton 2021 [76]	1091	53.2	−	−	−	−	−	−	−	−	−
Teshale 2020 [77]	531	−	−	−	−	−	64.6	−	−	−	−
Bernard Marc 2023 [78]	193	−	91.7	−	−	−	−	−	−	−	−
Chagomerana 2023 [79]	291	−	−	−	−	85.3	−	−	−	−	−
Dorward 2023 [80]	22,821	−	63.6	−	−	−	−	−	−	−	−
Gemechu 2023 [81]	235	68.5	−	−	−	−	−	−	−	−	−
Govere 2023 [82]	403	66.3	−	−	−	−	−	−	−	−	−
Hamooya 2023 [83]	3649	91.6	92.1	−	92.1	−	−	−	−	−	−
Joaquim 2023 [84]	1247	−	75.1	−	−	−	−	−	−	−	−
Kimanga 2023 [85]	7046	69.0	−	−	−	−	−	−	−	−	−
Lavoie 2023 [86]	75,348	−	58.0	−	−	−	−	−	−	−	−
MacKellar 2022 [87]	769	40.1	47.1	50.5	−	−	−	−	−	−	−
Masaba 2023 [88]	1515	88.4	89.0	−	−	−	−	−	−	−	−
Masuke 2023 [89]	9025	−	90.0	−	−	−	−	−	−	−	−
Mody 2021 [90]	65,673	−	59.9	−	−	−	−	−	−	−	−
Mugenyi 2022 [20]	20,171	77.0	−	−	−	−	−	−	−	−	−
Mwamuye 2022 [91]	786	76.5	71.0	65.6	60.8	−	−	−	−	−	−
Nimwesiga 2023 [92]	80	−	−	35.0	−	−	−	−	−	−	−
Ojiambo 2023 [93]	328	43.3	35.7	−	−	−	−	−	−	−	−
Ross 2023 [94]	29,017	−	64.3	−	−	−	−	−	−	−	−
Singh 2023 [95]	135	91.9	−	−	−	−	−	−	−	−	−
Tlhajoane 2021 [36]	829	−	47.9	−	−	−	−	−	−	−	−
Zhong 2023 [96]	285	−	83.9	−	−	−	−	−	−	−	−
Maskew 2024 [97]	35,830	72.5	61.3	−	−	−	−	−	−	−	−
Xia 2024 [98]	4688	−	95.3	−	−	−	−	−	−	−	−
Bekele 2024 [99]	1229	76.0	71.8	−	−	−	−	−	−	−	−
Tekpa 2024 [100]	5508	−	−	−	59.2	−	−	−	−	−	−
Badejo 2020 [101]	12,357	−	58.0	−	−	−	−	−	−	−	−
Brazier 2024 [102]	9002	−	−	−	−	45.6	−	−	−	−	−
Simple average		73.1	72.6	61.7	73.2	67.7	64.8	82.8	74.7	−	−
Weighted average^c^, ^d^		74.2	66.5	93.8	79.9	74.2	75.7	76.3	74.7	−	−
Weighted average interpolated^c^, ^e^		81.9	69.5	89.8	84.0	74.5	75.0	76.3	74.7	−	−

Abbreviation: ART, anti‐retroviral therapy.

“−” indicates that these results could not be determined from the report.

^a^ Includes 20 participants who are 10–19 years of age.

^b^ Includes 37 participants who are < 20 years of age.

^c^ Weighted by cohort size.

^d^ Weighted based on reported data.

^e^ Fewer studies report to later time points; therefore, pooled estimates at these time points can increase despite using interpolated data.

**Table 3:** Retention of participants 6, 12, 18, 24, 36, 48, 60 and 72 months after ART initiation calculated according to the data reported by each study included in a systematic review of retention in care among those on antiretroviral therapy in low‐ and middle‐income countries from 2015 to 2024, stratified by country

		Percentage of participants retained at month:							
Country	*N*	6	12	18	24	36	48	60	72
**Africa** ^a^									
Botswana	1523		95.9						
Burundi	29,829		97.7		93.5	87.3	81.0	75.9	74.7
Cameroon	423				73.0				
Central African Republic	5508				59.2				
Democratic Republic of Congo	11,281	89.0	83.0		76.0	69.0	67.0		
Eswatini	769	40.1	47.1	50.5					
Ethiopia	5026	76.9	73.5				64.6		
Kenya	18,106	76.6	78.9	65.6	60.8				
Malawi	2874	53.2	74.7			87.6			
Mozambique	1247		75.1						
Nigeria	94,562	63.9	59.8		55.4	51.2	46.7		
Rwanda	1082	51.7							
Senegal	207		59.9						
South Africa	100,676	72.1	67.4						
Tanzania	9927		74.8						
Uganda	34,516	68.5	63.9	35.0	75.3				
Zambia	101,519	91.6	76.0	95.6	92.1				
Zimbabwe	1523	76.7	60.1		91.8				
*Regional average*	420,598	69.1	72.5	61.7	75.2	73.8	64.8	75.9	74.7
**Latin America and the Caribbean**									
Haiti	193		91.7						
*Regional average*	193		91.7						
**Europe**									
Ukraine	1057		90.5						
*Regional average*	1057		90.5						
**Asia**									
Afghanistan	124		94.4						
China	4973		89.6						
India	135	91.9							
Indonesia	606	75.4							
Thailand	2354	82.5	84.0						
Vietnam	4892		92.2						
*Regional average*	8396	83.3	90.0						

Abbreviation: ART, anti‐retroviral therapy.

^a^ Three studies (Romo et al. [70], Ross et al., [94] and Brazier et al., [102]) are excluded from these estimates, as we were unable to determine retention estimates for each country included in the study.

**Figure 3:**
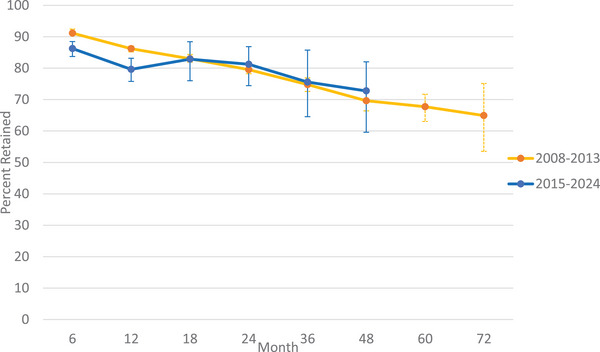
Comparison of pooled retention estimates in the pre‐UTT (2008−2013) and UTT (2015−2023) era.^1,2^ Abbreviations: pre‐UTT, pre‐Universal Test and Treat; UTT, Universal Test and Treat. ^1^Estimates from pre‐UTT were obtained from our prior systematic review conducted in 2015. ^2^Estimates at 60 and 72 months are not reported for the UTT era given the very few studies that reported data to these time points.

Table 3 presents simple average retention rates by country and region, utilizing only the reported data (i.e. no interpolation) with the majority of countries from Africa. Simple average retention at 12 months was 72.5% for Africa, 91.7% in the Caribbean, 90.5% in Europe and 90.0% in Asia. However, the European region only included Ukraine, and the Caribbean region only included Haiti. In Figure 2, we illustrate retention rates and their corresponding 95% CIs at each time point up to 36 months, utilizing interpolated data for each study, as well as the pooled estimate from the GLMM model (Figure S1 provides estimates at 18, 48 and 60 months). The pooled estimates from the GLMM model were 86.2% (95% CI: 83.7, 88.8) at 6 months, 79.6% (95% CI: 75.8, 83.2) at 12 months, 81.2% (95% CI: 74.4, 86.9) at 24 months and 75.6% (95% CI: 64.6, 85.8) at 36 months. When excluding studies that used data in 2015, the pooled estimate at 6 months was 84.8% (95% CI: 81.3, 87.7), at 12 months was 79.2% (95% CI: 74.1, 83.7), at 24 months was 78.0% (95% CI: 68.4, 85.2) and at 36 months was 68.9% (95% CI: 48.4, 85.2). Compared to our 2015 retention review, retention was slightly lower during the UTT period compared to the pre‐UTT era (Figure 3).

Figure 2Forest plots of calculated percent retained their corresponding 95% CIs by time point using data reported by each study included in a systematic review of retention in care among those on antiretroviral therapy in low‐ and middle‐income countries from 2015 to 2024 at 6, 12, 24 and 36 months.

**Figure:**
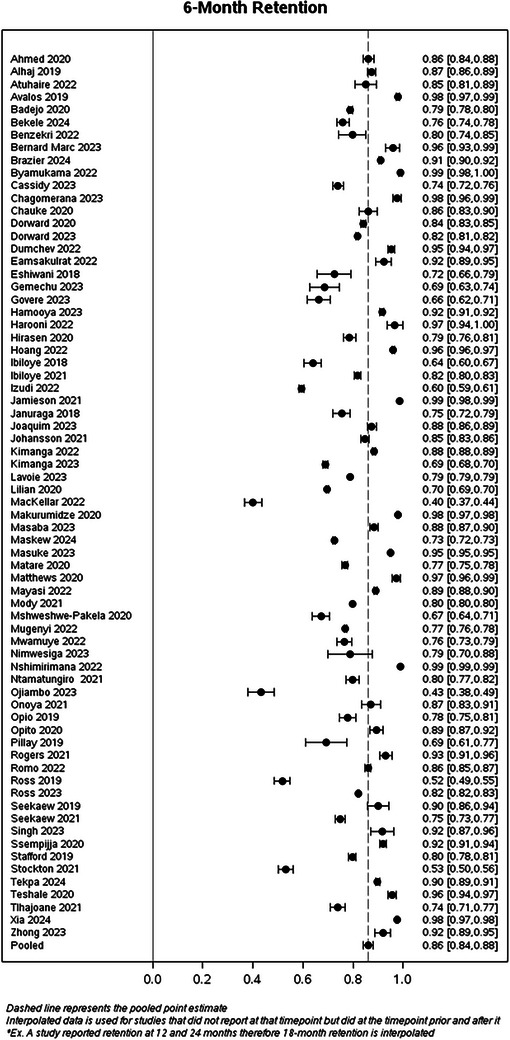

**Figure:**
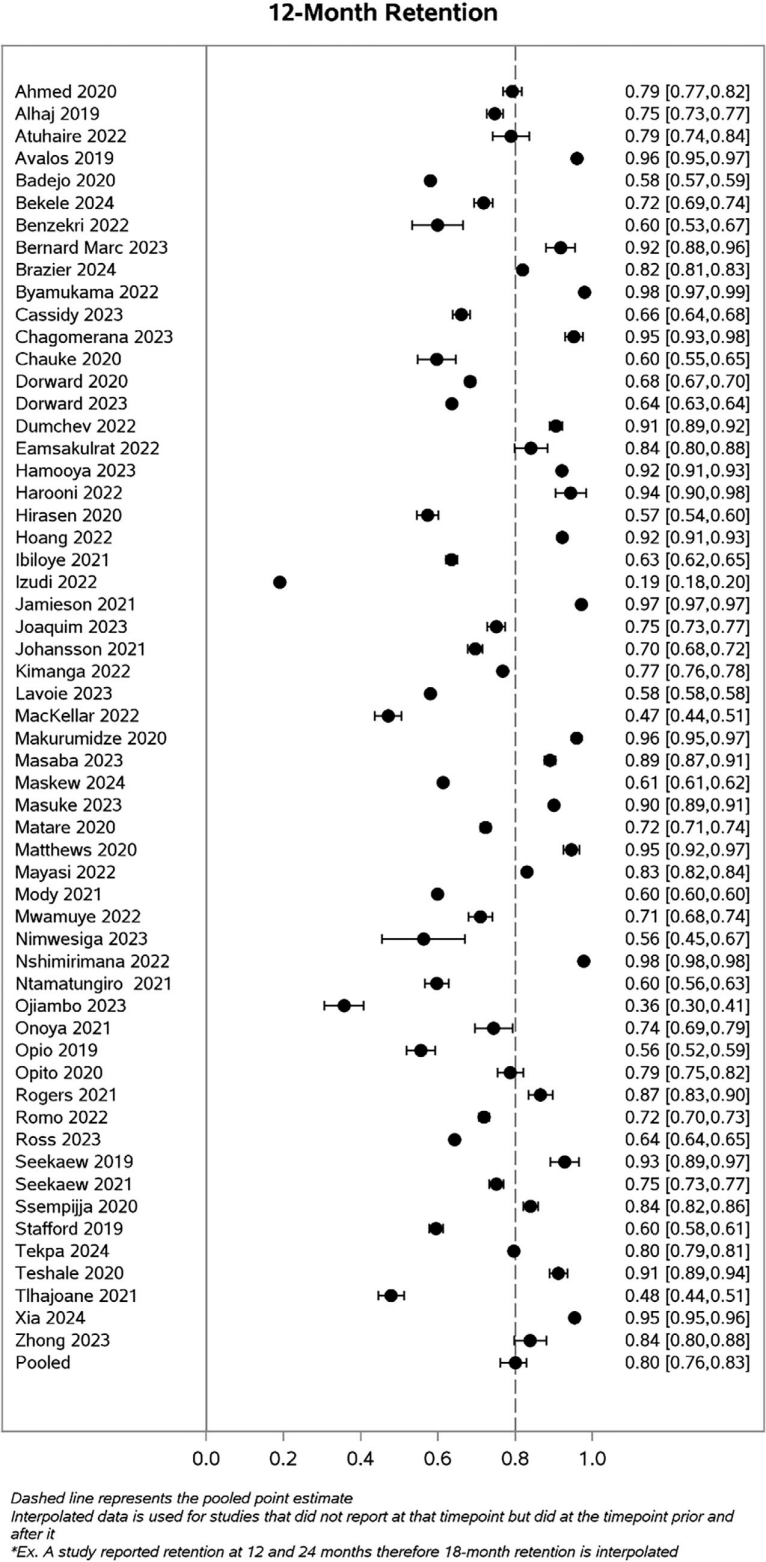

**Figure:**
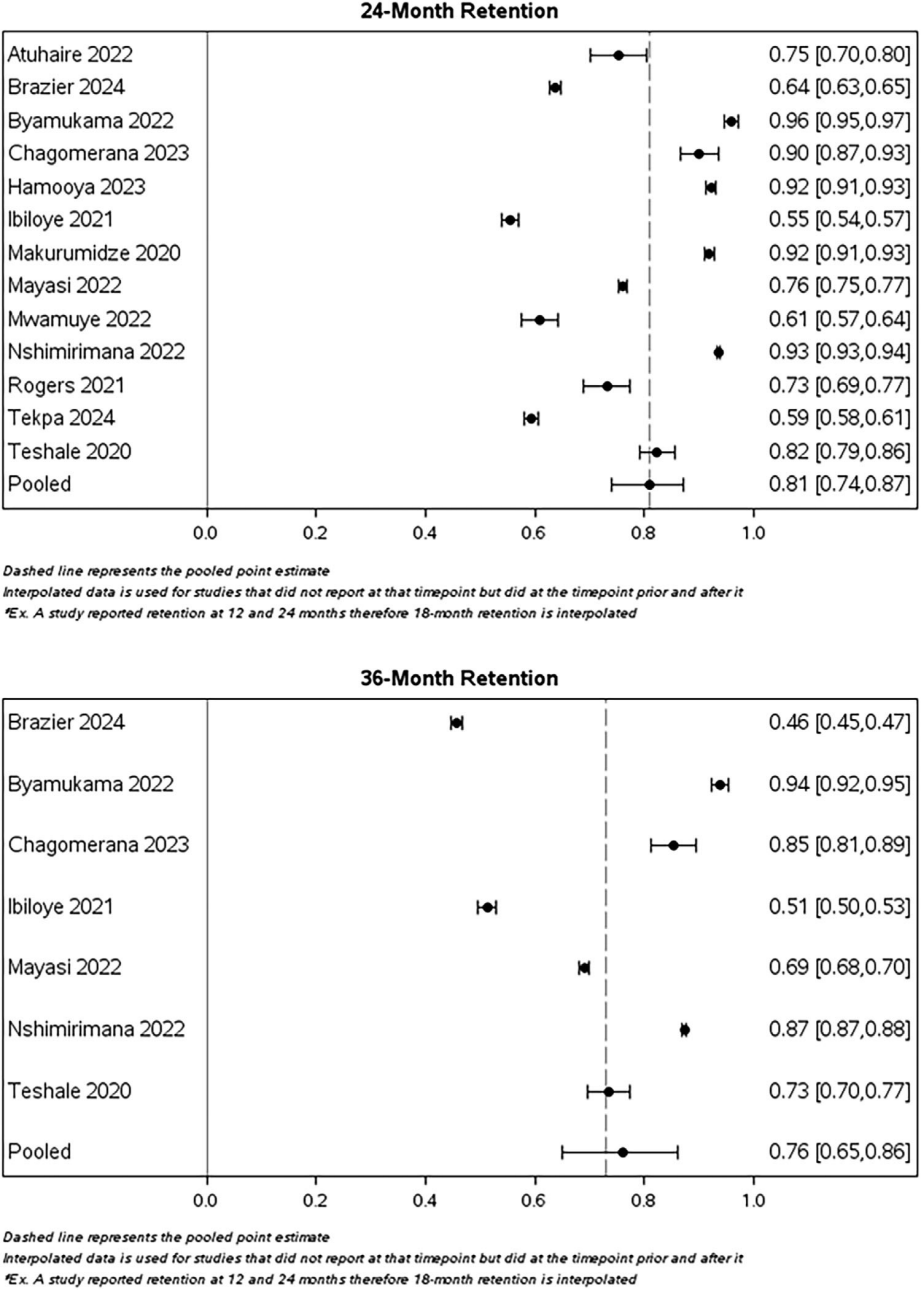

Table 4 presents the percent of patients who had a documented death, transfer, and disengaged from care for each study and overall. A weighted average of 1.9% of all participants who initiated ART had a documented death, 7.7% had a documented transfer, 27.3% were lost to follow‐up by the end of the reported follow‐up period, with a weighted average for all‐cause attrition at 32.6% (Table 4). Among the nine studies that reported treatment stoppage, an unweighted average of 2.1% of patients stopped treatment. Among the four studies that reported on interruptions, an unweighted average of 9.6% of patients reported any treatment interruption.

**Table 4:** Median follow‐up, all‐cause attrition and retention calculated according to the data reported by each study included in a systematic review of retention in care among those on antiretroviral therapy in low‐ and middle‐income countries from 2015 to 2024

Reference	Median follow‐up (months)	Died (A)	Lost to follow‐up (B)	Transferred care (C)	Total attrition from original site (D)^a^	Total retained at original site (E)^a^
Ahmed 2020 [38]	12.0	5.7	15.1	−	20.8	79.2
Alhaj 2019^b^ [39]	12.5	3.2	14.5	7.4	25.1	74.9
Atuhaire 2022 [40]	24.0	0.4	26.5	10.5	26.5	73.5
Avalos 2019 [41]	12.0	1.9	2.2	−	4.1	95.9
Benzekri 2022 [42]	12.0	14.5	15.9	3.9	34.3	65.7
Byamukama 2022^c^ [32]	34.8	2.9	2.4	5.1	10.4	89.6
Cassidy 2023 [43]	9.0	−	34	−	34.0	66.0
Chauke 2020 [44]	12.0	−	28.4	12	40.4	59.6
Dorward 2020^c^ [33]	9.2	0.5	20.4	6.1	27.0	73.0
Dumchev 2022 [45]	12.0	−	−	−	9.5	90.5
Eamsakulrat 2022 [46]	12.0	3	7.8	5.2	15.9	84.1
Ntamatungiro 2021 [47]	12.0	3.7	27.7	8.8	40.1	59.9
Eshiwani 2018 [48]	6.0	0	27.5	0	27.5	72.5
Harooni 2022 [49]	12.0	−	5.6	−	5.6	94.4
Hirasen 2020 [50]	12.0	2.6	17.2	22.8	42.6	57.4
Hoang 2022 [51]	12.0	−	7.8	−	7.8	92.2
Ibiloye 2018 [37]	7.4	3.4	23.4	9.3	36.1	63.9
Ibiloye 2021 [52]	30.0	1.2	46	−	47.2	52.8
Izudi 2022 [53]	12.0	3.3	77.6	−	80.9	19.1
Jamieson 2021 [54]	18.3	0.13	4.2	−	4.3	95.7
Januraga 2018 [55]	6.0	1.5	24.3	6.3	32.1	67.9
Johansson 2021 [56]	12.0	6.3	24.1	−	30.4	69.6
Kimanga 2022 [57]	12.0	3.1	20.3	−	23.3	76.7
Lilian 2020 [58]	6.0	0.6	24.2	5.5	30.3	69.7
Makurumidze 2020^d^ [59]	17.7	−	−	−	8.2	91.8
Matare 2020 [60]	12.0	0.08	27.5	−	27.6	72.4
Matthews 2020 [61]	8.2	0.9	4.6	−	5.5	94.5
Mayasi 2022 [62]	25.0	−	−	−	33.0	67.0
Mshweshwe‐Pakela 2020 [63]	6.0	−	32.8	−	32.8	67.2
Nshimirimana 2022 [64]	42.0	3.9	25.3	8.3	25.3	74.7
Onoya 2020 [65]	12.0	−	34.4	−	34.4	65.6
Opio 2019 [66]	*	2.5	33.4	8.5	44.4	55.6
Opito 2020^e^ [67]	12.0	1.3	12.7	7.3	21.3	78.7
Pillay 2019 [68]	6.0	−	31	−	31.0	69.0
Ajeh 2021 [69]	24.0	2.6	17.3	7.1	27.0	73.0
Romo 2022 [70]	12.0	7.9	15.2	−	23.2	76.8
Ross 2019 [71]	9.0	1	42.7	4.6	48.3	51.7
Seekaew 2019 [72]	9.0	−	7.1	−	7.1	92.9
Seekaew 2021 [73]	9.0	0.4	24.1	−	24.5	75.5
Ssempijja 2020 [74]	12.0	2	10	4	16.0	84.0
Stafford 2019 [75]	12.0	2	34	5	41.0	59.0
Stockton 2021 [76]	6.0	0.2	−	6.6	6.8	93.2
Teshale 2020 [77]	19.6	9.6	19.21	6.6	35.4	64.6
Bernard Marc 2023 [78]	12.0	1.6	6.7	−	8.3	91.7
Chagomerana 2023 [79]	36.0	2.3	5	4.7	12.0	88.0
Dorward 2023 [80]	12.0	1.1	21.1	14.2	36.4	63.6
Gemechu 2023 [81]	6.0	5.9	16.6	8.9	31.5	68.5
Govere 2023 [82]	6.0	4.22	28.04	1.5	33.8	66.3
Hamooya 2023 [83]	24.0	0.2	5.9	3.2	9.3	90.7
Joaquim 2023 [84]	12.0	−	24.9	−	24.9	75.1
Kimanga 2023 [85]	6.0	1.7	24.5	4.8	31.0	69.0
Lavoie 2023 [86]	12.0	1.5	35.8	4.8	42.0	58.0
MacKellar 2022 [87]	31.6	1.8	43	9.8	54.6	45.4
Masaba 2023 [88]	11.9	0.4	1.8	1.1	3.3	96.7
Masuke 2023 [89]	12.0	−	−	−	10.0	90.0
Mody 2021 [90]	12.0	−	40.1	−	40.1	59.9
Mugenyi 2022 [20]	6.0	2.6	20.4	−	23.0	77.0
Mwamuye 2022 [91]	24.0	3.8	30.3	9.5	43.6	56.4
Nimwesiga 2023 [92]	9.0	1.2	18.8	45	65.0	35.0
Ojiambo 2023 [93]	9.0	9.1	55.2	−	64.3	35.7
Ross 2023 [94]	12.0	3.8	25.1	6.7	35.7	64.3
Singh 2023 [95]	6.0	−	8.1	−	8.1	91.9
Tlhajoane 2021 [36]	6.0	4.5	22.7	8.3	35.5	64.5
Zhong 2023 [96]	12.0	3.9	0.7	−	4.6	95.4
Maskew 2024 [97]	12.0	1.5	22.2	−	23.7	76.3
Xia 2024 [98]	12.0	−	4.7	−	4.7	95.3
Bekele 2024 [99]	12.0	4.8	15.9	−	20.7	79.3
Tekpa 2024 [100]	12.0	−	40.8	−	40.8	59.2
Badejo 2020 [101]	12.0	1.2	24.2	8.1	25.4	74.6
Brazier 2024 [102]	18.0	−	−	−	54.4	45.6
Simple averages^b^	13.5	2.9	22.0	8.1	28.0	72.0
Weighted averages^b^, ^c^	14.2	1.9	27.3	7.7	32.6	67.4

*Median follow‐up period by group was reported as: Enrolled in 2016: 24.1 and Enrolled in 2017: 16.6; A collapsed value could not be calculated as *N*s by group were not reported.

“−” indicates that these data could not be determined from the report.

^a^ Calculation: D = A+B+C; D = 1 – C.

^b^ Death, transfer and lost to follow‐up averages are lower than expected, as not every study reported each group and some only reported all‐cause attrition, which included a combination of death and lost to follow‐up.

^c^ Weighted by cohort size.

We conducted a sensitivity analysis where we modelled attrition under the best‐case, worst‐case and midpoint scenarios, as not all studies reported retention to the same time point (Figure S2). There is minimal difference in retention rates across the three scenarios in the first year of treatment. The difference widens at 18 months and continues to expand to 96 months. At 24 months, the weighted average midpoint scenario retention is 52.5% (best to worst‐case range 69.7% to 36.4%). Findings from this analysis differ from our prior reviews, where we found minimal difference in retention rates across the three scenarios in the first 24 months.

### 3.3 Quality assessment

The possible range of scores was −7 to 13, with a higher score indicating better quality. The average score for quality was 8.2 (Standard Deviation: 3.4), with the highest score being 13 and the lowest score being −1, indicating that overall study quality was moderate to high. A majority of studies included a clear and well‐defined definition of LTFU. Few studies clearly reported the median follow‐up time; therefore, follow‐up time was typically determined based on assumptions described in the methods section above. As a result, this question had the poorest score with a mean of −0.6. It is important to remember that we included abstracts from conferences as well as published studies, and abstracts tended to score on the lower end of the scale due to word limit constraints.

## 4 DISCUSSION

In this review of retention in the UTT era, capturing 84,427 participants from 35 countries, we estimated pooled retention to be 86.2% at 6 months, 79.6% at 12 months, 81.2% at 24 months, 75.6% at 36 months and 72.8% at 48 months. These results are largely similar but not identical to findings from our 2015 review, where retention was 91.0% at 6 months, 86.0% at 12 months, 79.0% at 24 months, 75% at 36 months and 69% at 48 months.

Findings from this updated review highlight a persistent and concerning trend of increasing attrition, especially after the first 2 years after ART initiation. Our estimated pooled retention rates in the first 12 months following ART initiation in the UTT era were lower compared to the 6‐ and 12‐month retention rates estimated in our prior review. While retention at 24 months was found to be slightly higher in the UTT era compared to in our prior review, retention after 24 months declined drastically and was lower, on average, than in our 2008–2013 estimates. However, the lower retention reported at these later time points should be interpreted with caution, given the small number of studies that report long‐term retention. Furthermore, we note that this comparison of pre‐UTT to post‐UTT is limited as we utilized data from our 2015 systematic review, and so the studies included in this review are not necessarily the same as the one from the 2015 review [8]. Additionally, it is important to consider that there were numerous changes in HIV care delivery post‐UTT, such as Same Day Initiation, and so these differences are not solely attributable to the implementation of UTT. These findings suggest that while UTT resulted in substantial increases in the number of people initiating ART and reduced time to ART initiation, it did not have a large and/or consistent impact on retention in care after ART initiation. However, we see stable retention as a positive outcome given the large increase in the number of patients initiating ART post‐UTT and given that those newly eligible under UTT may have less incentive to remain in care than those previously eligible at lower CD4 counts, as they likely were not experiencing disease‐related illness before treatment. We note that in the pre‐UTT era, patients who initiated ART had already demonstrated the willingness and ability to make multiple clinic visits and remain in HIV care. Patients who are initiating in the post‐UTT era may thus be different from the patients who returned to the clinic to initiate treatment in the pre‐UTT era [103].

Within our search, only five cohorts reported on retention beyond 36 months, and so the retention estimates reported at 36 months and beyond should be interpreted with caution. Similarly, retention by region should also be interpreted with caution given the few number of countries in some regions like Europe and Asia. There is a need for future studies to report on longer‐term retention to improve our ability to accurately estimate retention in case. While short‐term outcomes are important to evaluate, patients need to be engaged in care and adherent to lifelong ART in order to prevent viral rebound and onward transmission. Notably, retention rates beyond 36 months were concerningly low in our review. Enhanced reporting of long‐term retention with a concerted effort on differentiating between true LTFU and undocumented transfers is essential to not only accurately estimate retention in care but improve our understanding of retention barriers across the life course and ultimately identify areas for targeted interventions to improve treatment outcomes. We also note that many cohorts did not report outcomes by year of treatment initiation, which forced us to exclude them from this review; more comprehensive descriptions of published cohorts would improve our understanding of long‐term retention [104, 105, 106, 107, 108, 109, 110, 111, 112, 113, 114].

While our review included all LMICs, we found very few studies reporting retention outcomes in countries outside of sub‐Saharan Africa. Even within sub‐Saharan Africa, the majority of studies came from South Africa. While the majority of PLWH reside on the African continent, it is concerning that we found very few studies reporting on treatment outcomes in other regions. We identified no studies that met our inclusion criteria from Latin America and only one study each from the Caribbean (Haiti) and Europe (Ukraine). There were very few countries in Asia that had retention outcomes in the post‐UTT era, and the majority came from Thailand (*n* = 3). Limited reporting on retention from varying geographic regions limits our ability to fully understand the impact of the implementation of UTT within and across regions.

While there is a PEPFAR definition of LTFU, there was considerable variability in how each study defined LTFU and could include undisclosed treatment stoppage, transfer from care and/or death [26]. Prior studies have demonstrated that the definition of LTFU used can meaningfully change retention rates [13, 115, 116, 117]. For example, some cohorts considered LTFU to have occurred at the point their definition of LTFU was met, not allowing participants to return to care, while others allowed participants to return at a later time point. Furthermore, if patients have surplus medications and do not return to the clinic to pick up ART medication, they could be considered LTFU, even though they are on treatment, leading to underestimates in retention. This could contribute to our retention estimates remaining largely unchanged in the post‐UTT era compared to the pre‐UTT era, as the majority of the studies’ definition of LTFU did not allow participants to return to care. Traditional on/off LTFU definitions do not accurately portray the true nature of care engagement as many participants will disengage from care and then re‐engage months, if not years, later [13, 118, 119, 120, 121, 122, 123]. This pattern, known as the “side door” in the care cascade, is an important factor that is often not well‐characterized in the literature. In addition, participants reported as LTFU in one cohort may have transferred care to another without informing the sending clinic and may present as new participants to the new clinic. Prior research has demonstrated that undocumented transfers can lead to substantial underestimates of retention; thus, retention rates at a national level are often much higher and more representative [13, 103, 124]. This is also true of death, as there may be a number of undocumented deaths when clinical record data is the primary data source. Thus, the number of deaths and transfers reported in our review is likely an underestimate of the true number. Our retention estimates are, therefore, more representative of “retention at site,” and the true rate of retention within ART programmes may be much higher.

This review had several limitations. First, larger cohorts (e.g. South Africa, Zambia and Burundi) likely had a strong impact on our retention estimates, especially when using the interpolated data. Second, while our interpolated data assumed a linear trend between the time points, attrition is not always linear, especially in the first year of initiation. Any studies where we interpolated the 6‐ and 12‐month outcomes could, therefore, overestimate retention at the earlier time points. Third, although we were interested in understanding retention after UTT adoption, several additional guideline changes (e.g. introduction of dolutegravir for first‐line ART and DSD models) were also implemented during the study period. As such, observed retention trends reflect a variety of forces and not solely UTT [16]. Additionally, our review used data being reported in 2015, and for many countries, full adoption and implementation of UTT took place in 2016 and beyond; therefore, a small number of our studies may include data from when UTT was not fully implemented. We did, however, conduct a sensitivity analysis, excluding any studies reporting data from 2015, and our pooled estimates were very similar. Fourth, while we conducted snowball sampling as well as a hand search to identify any articles that were not MeSH‐indexed, some studies reporting on retention were likely missed because the terms utilized to index ART retention are not uniform across publications. The effect of this bias is unknown. Fifth, as few countries outside of the African region were identified, we were unable to evaluate differences across regions. Our findings are likely not indicative of retention outcomes in regions with concentrated epidemics and/or varying treatment guidelines. Sixth, COVID‐19 lockdowns and changes in clinic protocols in 2020 may have had an impact on the number of studies being published on retention and on long‐term retention outcomes. The direction of this effect may have varied with some patients more likely to experience treatment interruption due to travel restrictions and clinic closures, and others benefiting from pandemic‐driven innovations, such as 12‐month scripting of ART in South Africa [125, 126]. Furthermore, recent changes in US policy and funding are likely to impact retention, especially as funding for key populations such as men who have sex with men and for data capturers has either been reduced and/or eliminated. These changes could adversely impact retention over the short‐ and long‐term and make it more difficult to continue monitoring retention in care using routine clinic data. Seventh, we required studies to be published in English or Chinese, and so this may impact the comprehensiveness of studies included and could explain the limited number of studies we found from Latin America and the Caribbean.

## 5 CONCLUSIONS

In this systematic review, we estimated retention on site in the era of UTT to be 86.2% at 6 months, 79.6% at 12 months, 81.2% at 24 months, 75.6% at 36 months and 72.8% at 48 months, suggesting that attrition after the first 2 years in care remains a concern, and continued efforts should be made to ensure participants remain engaged in care over their lifetime. A recent review by Burke et al., based primarily on results from before the COVID‐19 pandemic, identified some of the most common reasons for disengagement from treatment in the UTT era, including medication side effects, socio‐economic factors such as employment, and the perception that ART medication lacks benefit [127]. These findings underscore barriers beyond access to ART and emphasize the need for multilevel interventions to prevent attrition and improve treatment outcomes [6, 128, 129]. Recent cuts to PEPFAR may impact retention in care as well as the quality of data to monitor retention. Future research is needed to assess the impact of these funding cuts on retention rates and data quality.

## COMPETING INTERESTS

All authors declare no conflict of interest.

## AUTHOR CONTRIBUTIONS

AZ, DBF, MPF and SR were involved in the conceptualization and design of the study. AZ and EMK acquired the data. AZ conducted the statistical analysis with support from MPF and SR. AZ wrote the first draft of the manuscript. All authors have full access to all the data in the study. All authors critically interpreted the results and developed the report. All authors reviewed and approved the final version. All authors are responsible for the decision to submit for publication.

## FUNDING

ATB and EK were supported by the National Institute of Diabetes and Digestive and Kidney Diseases K01DK116929. SR was supported by INV‐031690 from the Gates Foundation to Boston University. AZ was supported by the National Institute of Allergy and Infectious Diseases F31AI179292. Research reported in this publication is supported by the National Institute of Mental Health of the National Institutes of Health under Award Number R01MH121998.

## DISCLAIMER

The content is solely the responsibility of the authors and does not necessarily represent the official views of the National Institutes of Health. The funders had no role in study design, data collection and analysis, decision to publish or preparation of the manuscript.

## Supporting information




**Figure S1**. Forest plots of calculated percent retained and their corresponding 95% CIs by time point using data reported by each study included in a systematic review of retention in care among those on antiretroviral therapy in low‐ and middle‐income countries from 2015 to 2024 at 18‐, 48‐, and 60‐months
**Figure S2**. Sensitivity analysis of best case, midpoint, and worst‐case scenarios of attrition based on studies included in a systematic review of retention in care among those on antiretroviral therapy in low‐ and middle‐income countries from 2015 to 2024^1^
**Table S1**. Search terms used to identify studies for evaluation to be included in a systematic review of retention in care among those on antiretroviral therapy in low‐ and middle‐income countries from 2015 to 2024
**Table S2**. Overview of the modifications made to the Joanna Briggs Institute Critical Appraisal Tool for Prevalence Studies for a systematic review on retention in care among those on antiretroviral therapy in low‐ and middle‐income countries from 2015 to 2024
**Table S3**. Characteristics of studies included in a systematic review of retention in care among those on antiretroviral therapy in low‐ and middle‐income countries from 2015 to 2024
**Table S4**. Definitions of loss to follow‐up as reported by each study included in a systematic review of retention in care among those on antiretroviral therapy in low‐ and middle‐income countries from 2015 to 2024

## Data Availability

The data that support the findings of this study are available from the corresponding author upon reasonable request.
